# Supplementary material for: Structural basis for transcription initiation by bacterial ECF σ factors
Source: Nat Commun. 2019 Mar 11;10:1153. doi: 10.1038/s41467-019-09096-y (PMC6411747; doi:10.1038/s41467-019-09096-y)
Supplement: Supplementary file 1 — Supplementary Information [file 41467_2019_9096_MOESM1_ESM.pdf]

Structural basis for transcription initiation by bacterial ECF σ factors

Lingting Li, Chengli Fang *et al.*

Supplementary Table 1. Plasmids used in this study.

REAGENT	SOURCE	REAGENT	SOURCE
pTolo-EX5	Tolo Biotech	pTolo-EX5-*Mtb*σ^{H}-S182A	This study
pTolo-EX5-*Mtb*σ^{H}	This study	pTolo-EX5-*Mtb*σ^{H}-R183A	This study
pTolo-EX5-*Mtb*σ^{H}-R46A	This study	pTolo-EX5-*Mtb*σ^{H}-H185A	This study
pTolo-EX5-*Mtb*σ^{H}-R49A	This study	pTolo-EX5-*Mtb*σ^{H}-R186A	This study
pTolo-EX5-*Mtb*σ^{H}-F72A	This study	pTolo-EX5-*Mtb*σ^{H}-R188A	This study
pTolo-EX5-*Mtb*σ^{H}-T76A	This study	pTolo-EX5-*Mtb*σ^{H}_{2}(1-95)	This study
pTolo-EX5-*Mtb*σ^{H}-N77A	This study	pTolo-EX5-*Mtb*σ^{H}-I85W/R84W	This study
pTolo-EX5-*Mtb*σ^{H}-K79A	This study	pTolo-EX5-*Mtb*σ^{H}_{4}(145-216)	This study
pTolo-EX5-*Mtb*σ^{H}-W81A	This study	pTolo-EX5-*Mtb*σ^{H}_{2}-DL-σ^{H}_{4}	This study
pTolo-EX5-*Mtb*σ^{H}-Y83A	This study	pTolo-EX5-*Mtb*σ^{H}_{2}-$\sigma^{E}_{3.2}$-σ^{H}_{4}	This study
pTolo-EX5-*Mtb*σ^{H}-R84A	This study	pTolo-EX5-*Mtb*σ^{H}_{2}-$\sigma^{L}_{3.2}$-σ^{H}_{4}	This study
pTolo-EX5-*Mtb*σ^{H}-R84W	This study	pTolo-EX5-*Mtb*σ^{H}_{2}-$\sigma^{M}_{3.2}$-σ^{H}_{4}	This study
pTolo-EX5-*Mtb*σ^{H}-I85A	This study	pACYCduet-*Mtb-rpo*A-*rpoZ*	This study
pTolo-EX5-*Mtb*σ^{H}-I85G	This study	pETduet-*Mtb-rpoB*(R282A)-*rpo*	This study
pTolo-EX5-*Mtb*σ^{H}-I85W	This study	pETduet-*Mtb-rpoB*(E285A)-*rpo*	This study
pTolo-EX5-*Mtb*σ^{H}-N88A	This study	pEASY-Blunt-p*ClpB*-spacer15	This study
pTolo-EX5-*Mtb*σ^{H}-I91A	This study	pEASY-Blunt-p*ClpB*-spacer16	This study
pTolo-EX5-*Mtb*σ^{H}-N92A	This study	pEASY-Blunt-p*ClpB*-spacer17	This study
pTolo-EX5-*Mtb*σ^{H}-K96A	This study	pEASY-Blunt-P*ClpB*-spacer18	This study
pTolo-EX5-*Mtb*σ^{H}-R99A	This study	pEASY-Blunt-p*ClpB*-spacer19	This study
pTolo-EX5-*Mtb*σ^{H}-Y166A	This study	pARTaq-N25-100-TR2-spacer1	This study
pTolo-EX5-*Mtb*σ^{H}-K167A	This study	pARTaq-N25-100-TR2-spacer1	This study
pTolo-EX5-*Mtb*σ^{H}-T179A	This study	pARTaq-N25-100-TR2-spacer1	This study
pTolo-EX5-*Mtb*σ^{H}-M181A	This study	pARTaq-N25-100-TR2-spacer1	This study
pEASY-Blunt-p*ClpB*(-5A)	This study	pEASY-Blunt-p*Rv2466c*	This study
pEASY-Blunt-p*ClpB*(-5T)	This study	pEASY-Blunt-p*ClpB*(-30A)	This study
pEASY-Blunt-p*ClpB*(-5G)	This study	pEASY-Blunt-p*ClpB*(-30T)	This study
pEASY-Blunt-p*ClpB*(-9A)	This study	pEASY-Blunt-p*ClpB*(-30G)	This study
pEASY-Blunt-p*ClpB*(-9G)	This study	pEASY-Blunt-p*ClpB*(-31T)	This study
pEASY-Blunt-p*ClpB*(-9C)	This study	pEASY-Blunt-p*ClpB*(-31G)	This study
pEASY-Blunt-p*ClpB*(-10A)	This study	pEASY-Blunt-p*ClpB*(-31C)	This study
pEASY-Blunt-p*ClpB*(-10G)	This study	pEASY-Blunt-p*ClpB*(-32G)	This study
pEASY-Blunt-p*ClpB*(-10C)	This study	pEASY-Blunt-p*ClpB*(-32C)	This study
pEASY-Blunt-p*ClpB*(-11A)	This study	pEASY-Blunt-p*ClpB*(-32A)	This study
pEASY-Blunt-p*ClpB*(-11T)	This study	pEASY-Blunt-p*ClpB*(-33A)	This study
pEASY-Blunt-p*ClpB*(-11C)	This study	pEASY-Blunt-p*ClpB*(-33T)	This study
pEASY-Blunt-p*ClpB*(-29G)	This study	pEASY-Blunt-p*ClpB*(-33C)	This study
pEASY-Blunt-p*ClpB*(-29T)	This study	pEASY-Blunt-p*ClpB*(-34A)	This study
pEASY-Blunt-p*ClpB*(-29C)	This study	pEASY-Blunt-p*ClpB*(-34T)	This study
		pEASY-Blunt-p*ClpB*(-34C)	This study

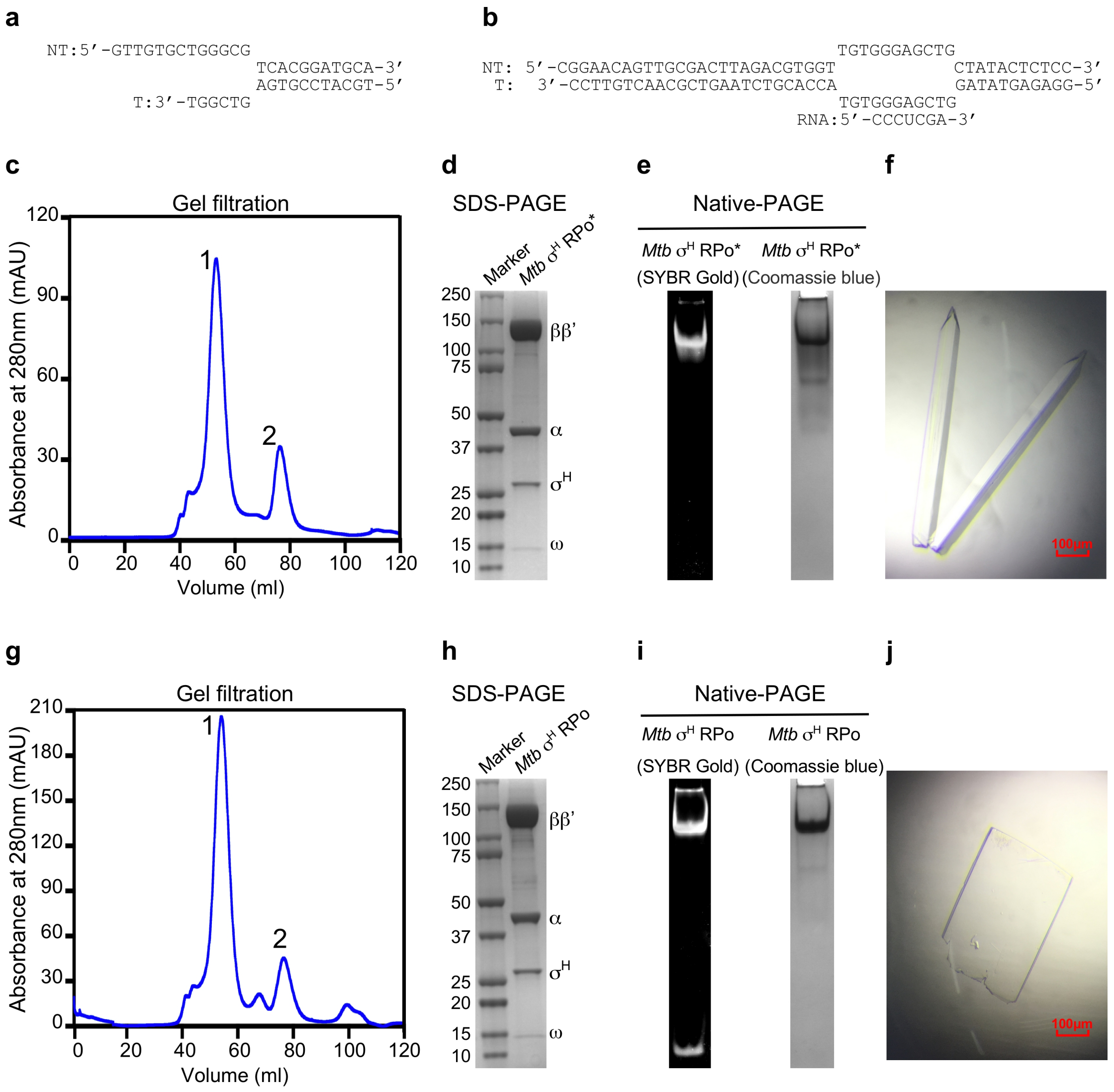

Supplementary Figure 1. Protein and crystals preparation of *Mtb* σ^{H}-RNAP holoenzyme and *Mtb* σ^{H}-RPo. Related to Figure 1 and 3. (a) DNA scaffold for preparation of σ^{H}-RPo* for crystallization of σ^{H}-RNAP holoenzyme. **(b)** Nucleic-acid DNA scaffold for preparation of σ^{H}-RPo. **(c)** Elution peaks of *Mtb* σ^{H}-RPo* from a size-exclusion column. Peak 1 is the *Mtb* σ^{H}-RPo* and peak 2 is the excess *Mtb* σ^{H}. **(d)** The SDS-PAGE of peak 1 in (C). **(e)** The Native-PAGE of peak 1 in (C). The gel was first stained with SYBR Gold for nucleic acids and then with Coomassie blue for protein. **(f)** The crystals of *Mtb* σ^{H}-holoenzyme. The DNA scaffold dissociated from RNAP during crystallization. **(g)** The elution peaks of *Mtb* σ^{H}-RPo from a size-exclusion column. Peak 1 is the *Mtb* σ^{H}-RPo and peak 2 is the excess *Mtb* σ^{H}. **(h)** The SDS-PAGE of peak 1 in (G). **(i)** The Native-PAGE of peak 1 in (G). The gels were stained as above. **(j)** The crystal of *Mtb* σ^{H}-RPo. * represents a RPo complex with a downstream fork promoter DNA.

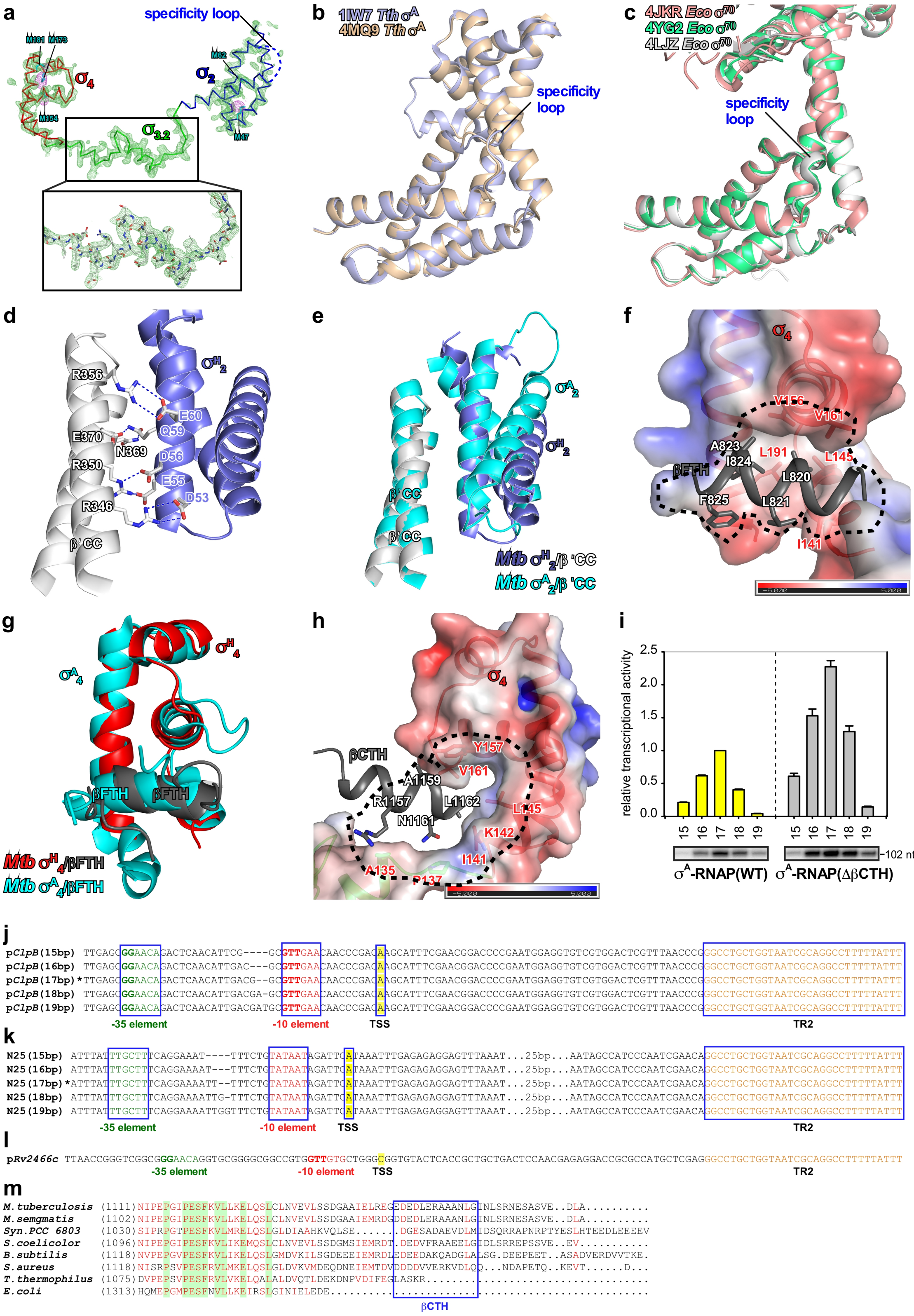

a
specificity loop
σ4
σ2
σ3.2
M181 M173
M154
M62
M47
b
1IW7 Tth σA
4MQ9 Tth σA
specificity loop
c
4JKR Eco σ70
4YG2 Eco σ70
4LJZ Eco σ70
specificity loop
d
R356
σH2
E60
E370
Q59
N369
D56
R350
E55
D53
R346
β'CC
e
σA2
σH2
β'CC
β'CC
Mtb σH2/β'CC
Mtb σA2/β'CC
f
σ4
V156
V161
A823
I824
L191
L145
βFTH
L820
F825
L821
I141
g
σH4
σA4
βFTH
βFTH
Mtb σH4/βFTH
Mtb σA4/βFTH
h
σ4
βCTH
Y157
A1159
V161
R1157
L1162
N1161
L145
K142
I141
A135
P137
i
relative transcriptional activity
2.5
2.0
1.5
1.0
0.5
0.0
15 16 17 18 19
15 16 17 18 19
102 nt
σA-RNAP(WT)
σA-RNAP(ΔβCTH)
j
pClpB(15bp) TTGAGCGGAACAGACTCAACATTCG----GCGTTGAACAACCCGACAAGCATTTCGAACGGACCCCGAATGGAGGTGTCGTGGACTCGTTTAACCCGGGCCTGCTGGTAATCGCAGGCCTTTTTATTT
pClpB(16bp) TTGAGCGGAACAGACTCAACATTGAC---GCGTTGAACAACCCGACAAGCATTTCGAACGGACCCCGAATGGAGGTGTCGTGGACTCGTTTAACCCGGGCCTGCTGGTAATCGCAGGCCTTTTTATTT
pClpB(17bp)*TTGAGCGGAACAGACTCAACATTGACG--GCGTTGAACAACCCGACAAGCATTTCGAACGGACCCCGAATGGAGGTGTCGTGGACTCGTTTAACCCGGGCCTGCTGGTAATCGCAGGCCTTTTTATTT
pClpB(18bp) TTGAGCGGAACAGACTCAACATTGACGA-GCGTTGAACAACCCGACAAGCATTTCGAACGGACCCCGAATGGAGGTGTCGTGGACTCGTTTAACCCGGGCCTGCTGGTAATCGCAGGCCTTTTTATTT
pClpB(19bp) TTGAGCGGAACAGACTCAACATTGACGATGCGTTGAACAACCCGACAAGCATTTCGAACGGACCCCGAATGGAGGTGTCGTGGACTCGTTTAACCCGGGCCTGCTGGTAATCGCAGGCCTTTTTATTT
-35 element
-10 element
TSS
TR2
k
N25(15bp) ATTTATTTGCTTTCAGGAAAT----TTTCTGTATAATAGATTCATAAATTTGAGAGAGGAGTTTAAAT...25bp...AATAGCCATCCCAATCGAACAGGCCTGCTGGTAATCGCAGGCCTTTTTATTT
N25(16bp) ATTTATTTGCTTTCAGGAAAAT---TTTCTGTATAATAGATTCATAAATTTGAGAGAGGAGTTTAAAT...25bp...AATAGCCATCCCAATCGAACAGGCCTGCTGGTAATCGCAGGCCTTTTTATTT
N25(17bp)*ATTTATTTGCTTTCAGGAAAATT--TTTCTGTATAATAGATTCATAAATTTGAGAGAGGAGTTTAAAT...25bp...AATAGCCATCCCAATCGAACAGGCCTGCTGGTAATCGCAGGCCTTTTTATTT
N25(18bp) ATTTATTTGCTTTCAGGAAAATTG-TTTCTGTATAATAGATTCATAAATTTGAGAGAGGAGTTTAAAT...25bp...AATAGCCATCCCAATCGAACAGGCCTGCTGGTAATCGCAGGCCTTTTTATTT
N25(19bp) ATTTATTTGCTTTCAGGAAAATTGGTTTCTGTATAATAGATTCATAAATTTGAGAGAGGAGTTTAAAT...25bp...AATAGCCATCCCAATCGAACAGGCCTGCTGGTAATCGCAGGCCTTTTTATTT
-35 element
-10 element
TSS
TR2
l
pRv2466c TTAACCGGGTCGGCGGGAACAGGTGCGGGGCGGCCGTGGTTGTGCTGGGCGGTGTACTCACCGCTGCTGACTCCAACGAGAGGACCGCGCCATGCTCGAGGGCCTGCTGGTAATCGCAGGCCTTTTTATTT
-35 element
-10 element
TSS
TR2
m
M.tuberculosis (1111)NIPEPGIPESFKVLLKELQSLCLNVEVLSSDGAAIELREGEDEDLERAAANLGINLSRNESASVE..DLA..........
M.semgmatis (1102)NIPEPGIPESFKVLLKELQSLCLNVEVLSSDGAAIEMRDGDDEDLERAAANLGINLSRNESASVE..DLA..........
Syn.PCC 6803 (1030)SIPRPGTPESFKVLMRELQSLGLDIAAHKVQLSE.......DGESADAEVDLMIDSQRRAPNRPTYESLHTEEDLEEEEV
S.coelicolor (1096)NIPEPGIPESFKVLIKEMQSLCLNVEVLSSDGMSIEMRDT.DEDVFRAAEELGIDLSRREPSSVE..EV...........
B.subtilis (1118)NVPEPGVPESFKVLIKELQSLGMDVKILSGDEEEIEMRDLEDEEDAKQADGLALSG.DEEPEET..ASADVERDVVTKE.
S.aureus (1118)NISRPSVPESFRVLMKELQSLGLDVKVMDEQDNEIEMTDVDDDDVVERKVDLQQ...NDAPETQ..KEVT......D...
T.thermophilus (1075)DVPEPSVPESFRVLVKELQALALDVQTLDEKDNPVDIFEGLASKR..
E.coli (1313)HQMEPGMPESFNVLLKEIRSLGINIELEDE...
βCTH

Supplementary Figure 2. The detailed interaction between *Mtb* RNAP core enzyme and σ^H. Related to Figure 1. (a) Electron density and model for σ^H. Green mesh, simulated-annealing Fo-Fc difference map with σ^H omitted contoured at 2.5 σ; Violet mesh, anomalous difference map contoured at 4 σ shows clear signals for 4 out of 5 Se atoms in σ^H. $\sigma^H{}_2$, blue; $\sigma^H{}_{3.2}$, green; $\sigma^H{}_4$, red. **(b)** The specificity loops adopt ordered conformation in *T. thermophilus* σ^A-RNAP holoenzymes (PDB: 1IW7 and 4MQ9) [https://www.rcsb.org/structure/1IW7; https://www.rcsb.org/structure/4MQ9]. **(c)** The specificity loops adopt ordered conformation in *E. coli* σ^{70}-RNAP holoenzymes (PDB: 4JKR, 4YG2, and 4LJZ) [https://www.rcsb.org/structure/4JKR; https://www.rcsb.org/structure/4YG2; https://www.rcsb.org/structure/4LJZ]. **(d)** The interaction between $\sigma^H{}_2$ (blue) and RNAP-β' coiled-coil (gray). H-bond, blue dash. **(e)** Superimposition of *Mtb* $\sigma^H{}_2$/β'CC (blue/gray) and *Mtb* $\sigma^A{}_2$/β'CC (cyan; PDB: 5UHA). **(f)** The interaction between $\sigma^H{}_4$ and RNAP βFTH. The electrostatic potential surface representation of $\sigma^H{}_4$ show a hydrophobic interface between $\sigma^H{}_4$ and RNAP βFTH. The electrostatic potential surface of $\sigma^H{}_4$ was generated using APBS tools in Pymol with partial changes determined by PDB2PQR server. Black dash, the hydrophobic groove; $\sigma^H{}_4$, red ribbon; RNAP βFTH, dark gray ribbon. **(g)** Superimposition of *Mtb* $\sigma^H{}_4$/βFTH (red/gray) and *Mtb* $\sigma^A{}_4$/βFTH (cyan; PDB: 5UHA). **(h)** The interaction between $\sigma^H{}_4$ and RNAP βCTH. The $\sigma^H{}_4$ electrostatic potential surface was generated as above. **(i)** The *in vitro* transcription activity of σ^A-RNAP(WT) holoenzyme (yellow bars) or σ^A-RNAP(ΔβCTH) holoenzymes (gray bars) from N25 promoter variants with -35/-10 spacer lengths of 15-19 base pairs. "102 nt" indicates length of terminated transcripts. The experiments were repeated for four times and the data were presented as mean ± S.E.M. **(j)** The promoter sequence of p*ClpB* derivatives for *in vitro* transcription activity of σ^H-RNAP(WT) and σ^H-RNAP(ΔβCTH). **(k)** The promoter sequence of N25 derivatives for *in vitro* transcription assay of σ^A-RNAP(WT) and σ^A-RNAP(ΔβCTH). **(l)** The promoter sequence of p*Rv2466c* for *in vitro* transcription assay of RNAP holoenzyme comprising σ^H derivatives. Source data of (I) are provided as a Source Data file. **(m)** a sequence alignment of RNAP βCTH of a few representative bacterial species.

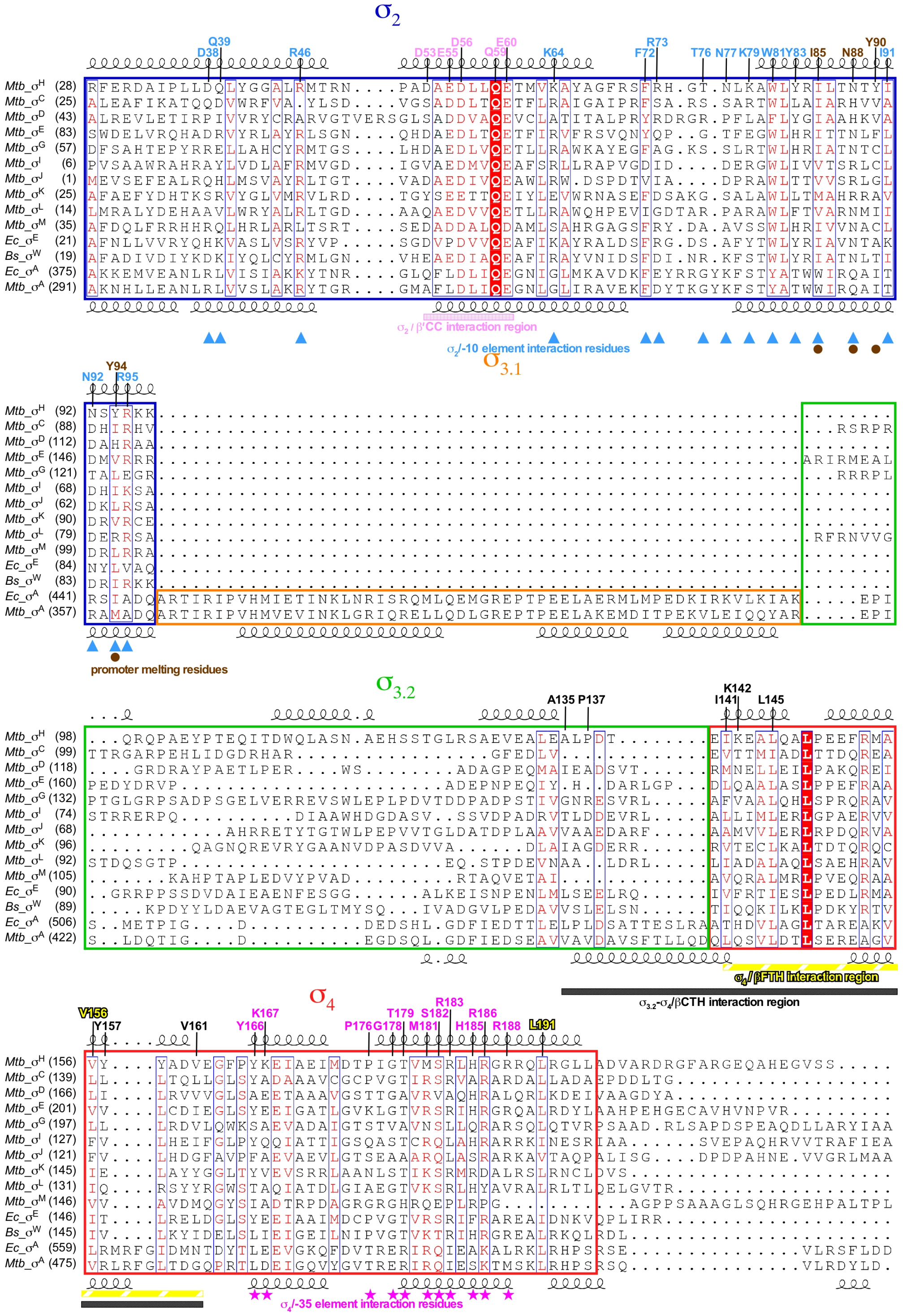

Supplementary Figure 3. The sequence alignment of the ECF σ factors and primary σ^A factors. Related to Figures 1-5. The regions of σ_2, $\sigma_{3.1}$, $\sigma_{3.2}$, and σ_4 were indicated by blue, orange, green, and red boxes, respectively. Residues contacting the promoter -10 element are indicated by blue filled triangles; residues participating in promoter melting are indicated by brown filled circles; residues contacting the -35 elements are indicated by violet filled stars. The RNAP-β'CC interacting region, RNAP-βFTH interacting region, and RNAP-βCTH region on σ_2 are highlighted by pink, yellow, and gray bars, respectively.

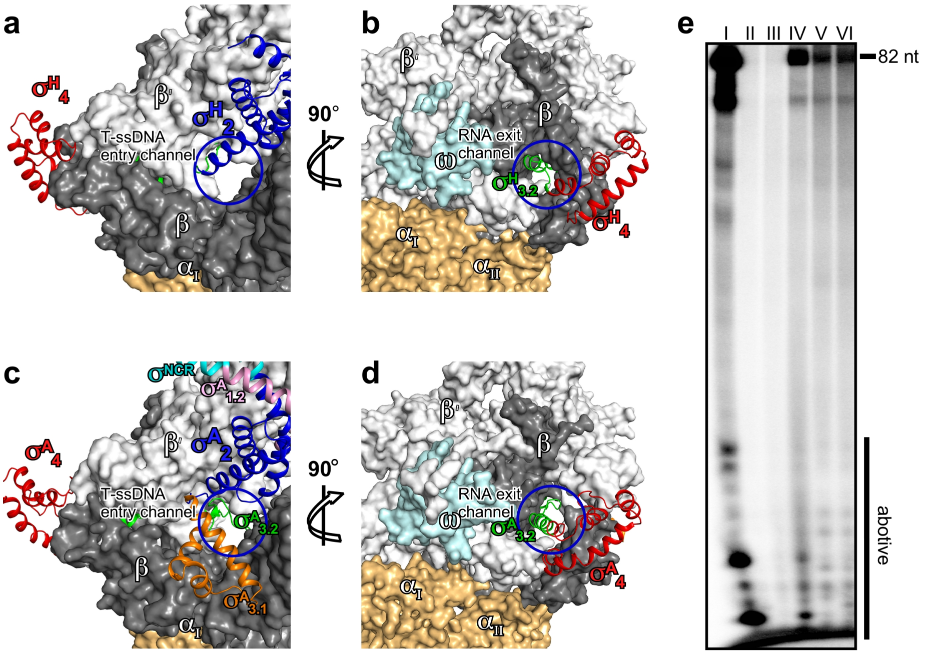

Supplementary Figure 4. The $\sigma_{3.2}$ pathway in *Mtb* σ^{H}-RNAP and σ^{A}-RNAP holoenzymes (extracted from PDB: 5UHA) [https://www.rcsb.org/structure/5UHA]. Related to Figure 2. (a) The entry channel of template single-strand DNA (T-ssDNA) created by σ^{H}_{2} and $\sigma^{H}_{3.2}$. **(b)** The RNA exit channel blocked by $\sigma^{H}_{3.2}$. **(c)** The entry channel of template single-strand DNA (T-ssDNA) created by σ^{A}_{2}, $\sigma^{A}_{3.1}$, and $\sigma^{A}_{3.2}$. **(d)** The RNA exit channel blocked by $\sigma^{A}_{3.2}$. RNAP core enzyme is shown as surface and σ is shown as ribbon. Colors are as in main figures. **(e)** The *in vitro* transcription activity from p*Rv2466c* promoter of RNAP holoenzymes comprising σ^{H} derivatives. "82 nt" indicates length of terminated RNA transcripts. "abortive" indicates abortive transcripts. I, II, III, IV, V, VI indicate wild type σ^{H} or σ^{H} derivatives same as in Figure 2D. Source data of (E) are provided as a Source Data file.

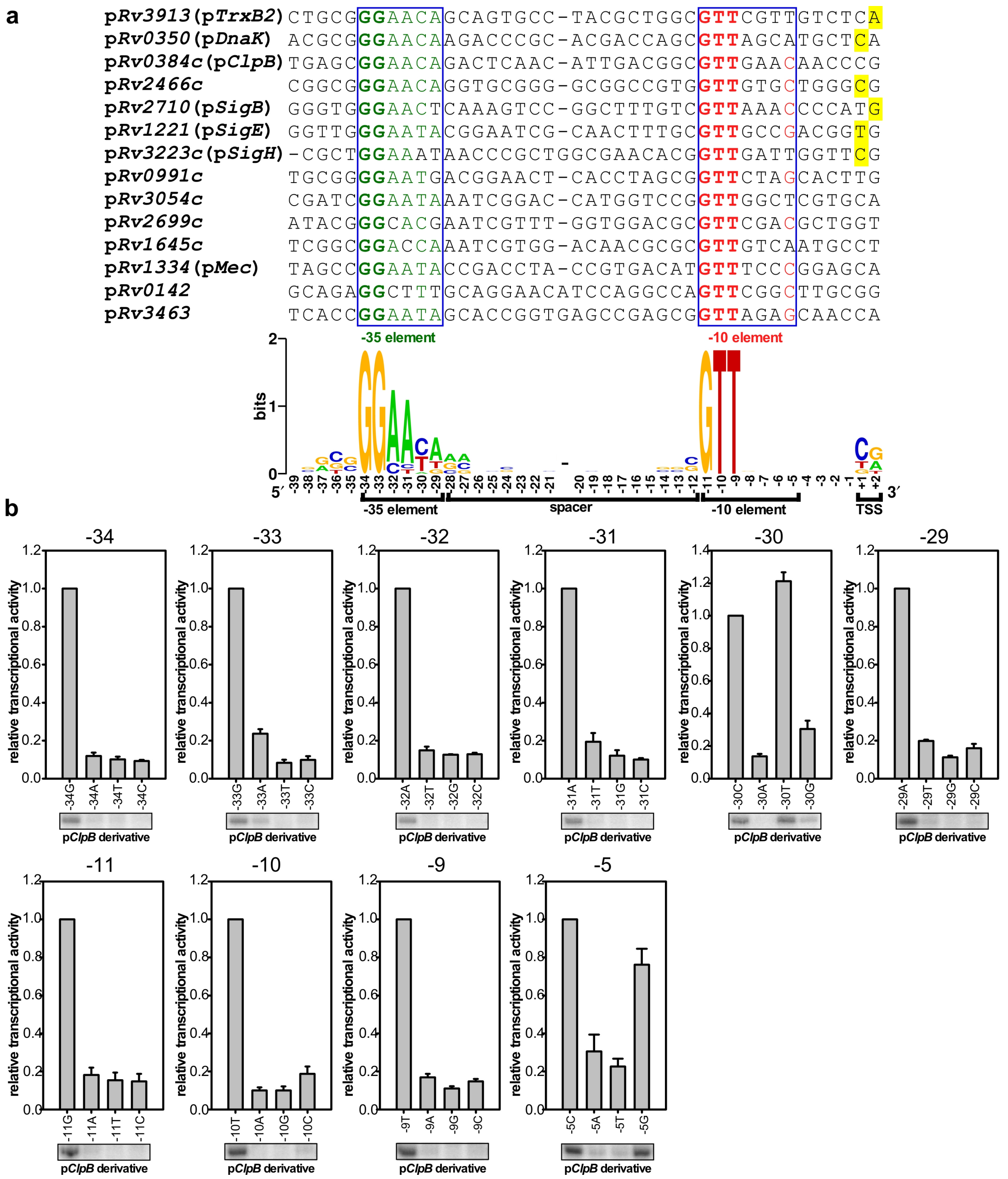

Supplementary Figure 5. The consensus sequence of *Mtb* σ^{H}-regulated promoters. Related to Figures 3-5. (a) The alignment of *Mtb* σ^{H}-regulated promoter sequences. The sequence logo was generated on the WebLogo server. The conserved -35 element, green; the conserved -10 element, red; the transcription start site (TSS), yellow. **(b)** The *in vitro* transcription assays showing specificity of the promoter -35 and -10 elements. The representative run-off (122 nt) products were showed and quantified. The experiments were repeated for three times and the data were presented as mean ± S.E.M. Source data of (B) are provided as a Source Data file.

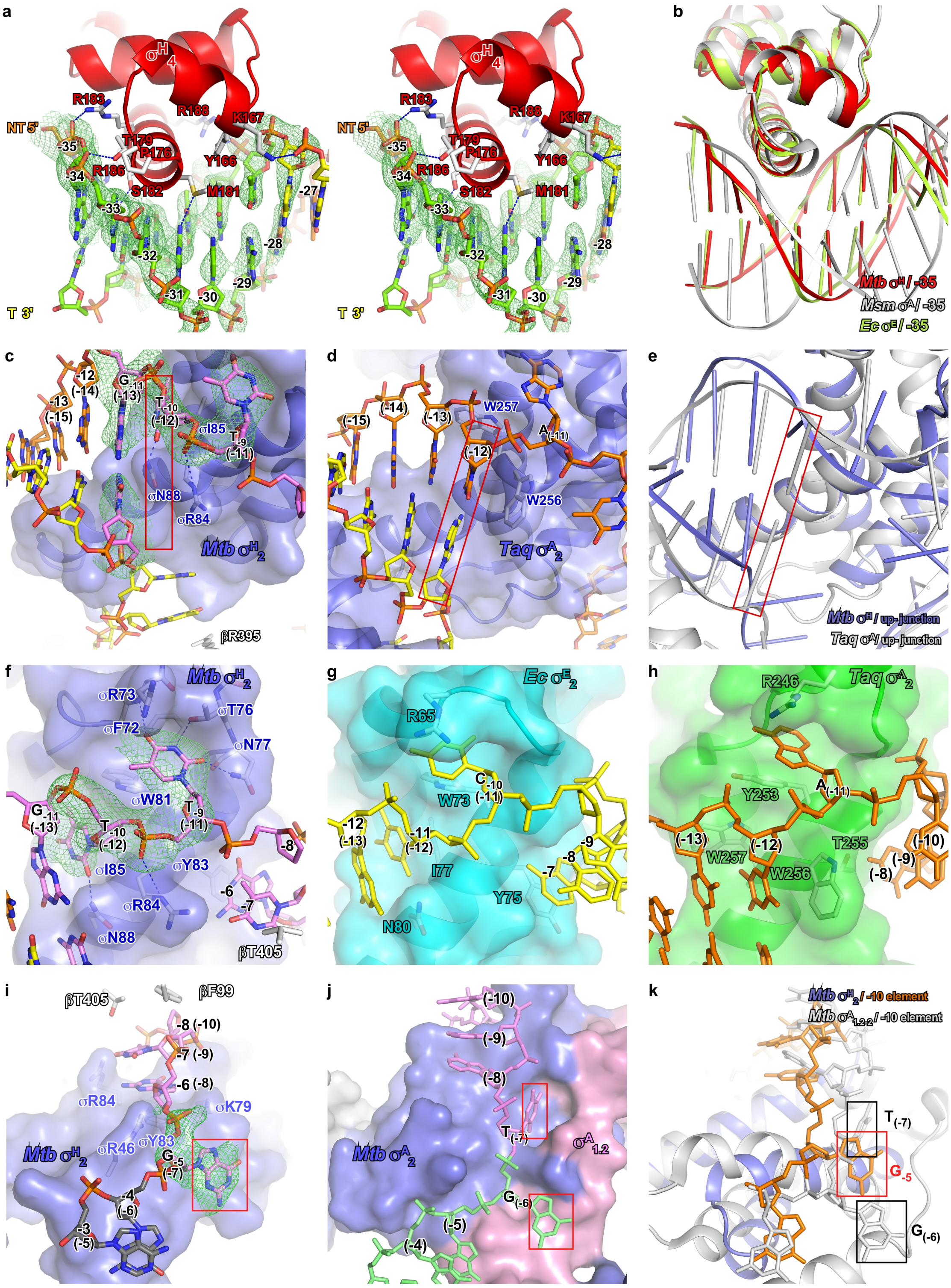

a
σH 4
R183
NT 5'
R188
K167
-35
T179
R176
R186
Y166
-34
S182
M181
-27
-33
-32
-28
-31
-30
-29
T 3'
b
Mtb σH / -35
Msm σA / -35
Ec σE / -35
c
-12 (-14)
G-11 (-13)
-13 (-15)
T-10 (-12)
σI85
T-9 (-11)
σN88
σR84
Mtb σH2
βR395
d
(-15)
(-14)
(-13)
W257
A(-11)
(-12)
W256
Taq σA2
e
Mtb σH / up-junction
Taq σA / up-junction
f
Mtb σH2
σR73
σT76
σF72
σN77
σW81
G-11 (-13)
T-10 (-12)
T-9 (-11)
-8
σI85
σY83
σR84
-6
-7
σN88
βT405
g
Ec σE2
R65
C-10 (-11)
W73
-12 (-13)
-11 (-12)
I77
-9
-7
-8
Y75
N80
h
Taq σA2
R246
A(-11)
Y253
(-13)
W257
(-12)
T255
(-10)
W256
(-9)
(-8)
i
βT405
βF99
-8 (-10)
-7 (-9)
-6 (-8)
σR84
σK79
Mtb σH2
σR46
σY83
G-5 (-7)
-4 (-6)
-3 (-5)
j
(-10)
(-9)
(-8)
T(-7)
σA1.2
Mtb σA2
G(-6)
(-5)
(-4)
k
Mtb σH2 / -10 element
Mtb σA1.2-2 / -10 element
T(-7)
G-5
G(-6)

Supplementary Figure 6. The detailed interactions of σ^H-RNAP with promoter DNA -35/-10 elements and the comparison with σ^A-RPo. Related to Figure 4. The *Mtb* σ^A-RPo structure was chosen for superimposition with *Mtb* σ^H-RPo unless the interactions to be compared is unavailable in *Mtb* σ^A-RPo. **(a)** The stereo presentation of detail interactions between σ$^H{}_4$ and the -35 dsDNA. Color as in Figure 4A. **(b)** The superimposition of *Mtb* σ$^H{}_4$/-35 element (red), *Ec* σ$^E{}_4$/-35 element (green; PDB: 2H27) [https://www.rcsb.org/structure/2H27] and *Msm* σ$^A{}_4$/-35 element (gray; PDB: 5TW1) [https://www.rcsb.org/structure/5TW1] structures. **(c)** σ$^H{}_2$ melts promoter DNA at the junction of -11/-10 (corresponding to (-13)/(-12) of promoters for the primary σ factor). **(d)** σ$^A{}_2$ melts promoter DNA at the junction of (-12)/(-11) (PDB: 4XLN) [https://www.rcsb.org/structure/4XLN]. **(e)** The comparison of promoter melting by *Mtb* σ^H-RNAP (blue) and *Taq* σ^A-RNAP (gray; PDB: 4XLN). **(f)** *Mtb* σ^H inserts the bases of $T_{(-12)}$(nt) and $T_{(-11)}$ (nt) into pockets. **(g)** *Ec* σ^E inserts the bases of $T_{(-12)}$(nt) and $C_{(-10)}$(nt) into pockets (PDB: 4LUP) [https://www.rcsb.org/structure/4LUP]. **(h)** *Taq* σ^A inserts the base of $A_{(-11)}$(NT) into a pocket (PDB: 4XLN). **(i)** Nucleotides at -7 and -6 positions of the -10 element ssDNA were stacked between βT405 and σY83 and G_{-5}(nt) is inserted into a pocket in σ^H-RPo. **(j)** Nucleotides at -10, -9 and -8 positions of the nontemplate DNA were stacked, $T_{(-7)}$(nt) of the -10 element and $G_{(-6)}$(nt) of the discriminator element are inserted into pockets in *Mtb* σ^A-RPo (PDB: 5UHA) [https://www.rcsb.org/structure/5UHA]. **(k)** The G_{-5}(nt) in σ^H-RPo (blue/orange) and $T_{(-7)}$(nt) in σ^A-RPo (gray; PDB:5UHA) are the last positions of the -10 element, respectively, and located on similar positions on σ. Green mesh, simulated-annealing Fo-Fc difference map with nucleic acids omitted contoured at 2.5 σ.

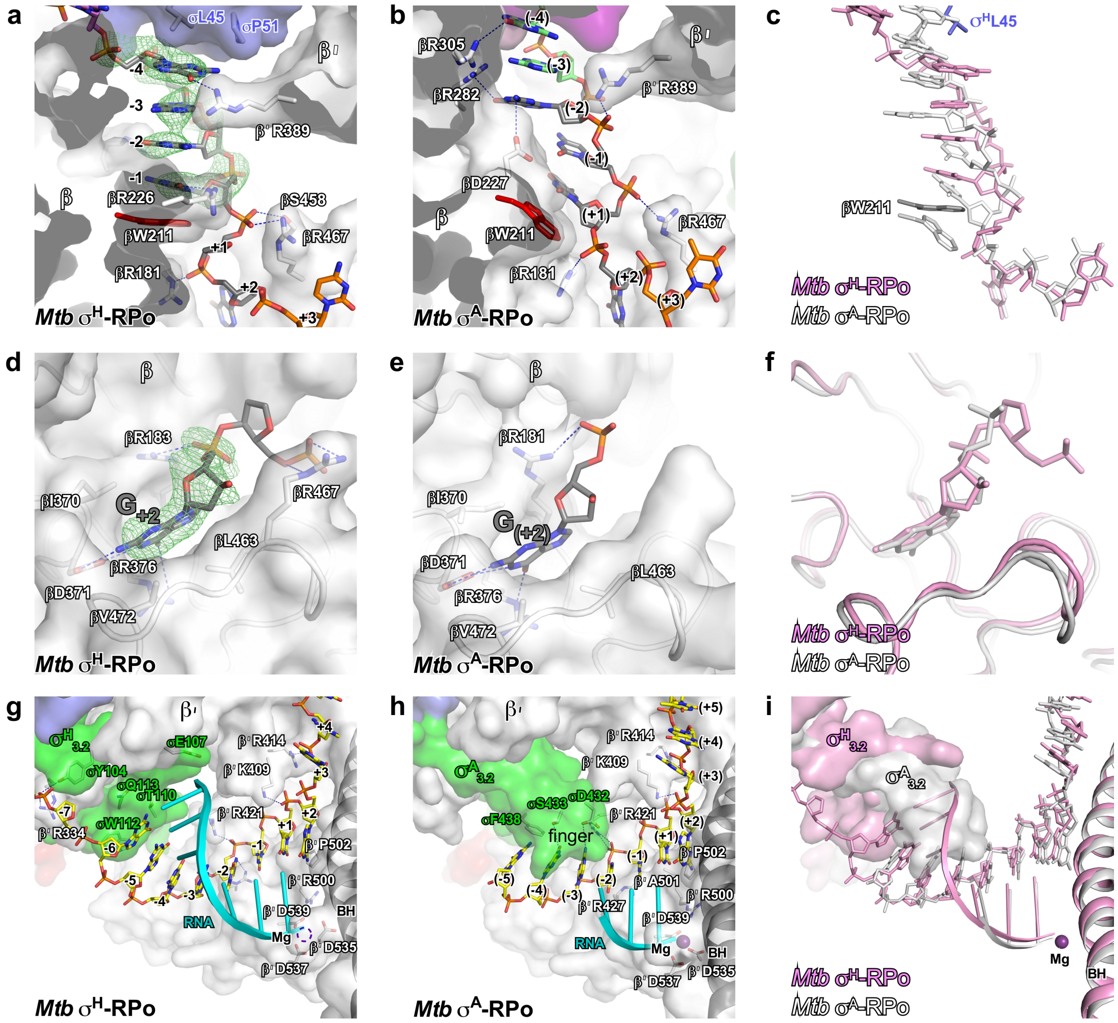

Supplementary Figure 7. The detailed interaction of σ^{H}-RNAP with CRE and DNA/RNA hybrid and the comparison with σ^{A}-RPo. Related to Figure 5. (a) Nucleotides from -4 to -1 positions of nontemplate CRE were sandwiched between σL45 and βW211 in σ^{H}-RPo. **(b)** The nucleotide from (-4) to (+1) positions of the nontemplate CRE were stacked by βW211 in *Mtb* σ^{A}-RPo (PDB: 5UHA) [https://www.rcsb.org/structure/5UHA]. **(c)** The structure superimposition of the CRE elements in *Mtb* σ^{H} -RPo (pink) and *Mtb* σ^{A}-RPo (gray; PDB: 5UHA). **(d)** The G$_{+2}$(nt) inserts into the 'G' pocket in σ^{H}-RPo. **(e)** The G$_{(+2)}$(nt) inserts into the 'G' pocket in *Mtb* σ^{A} -RPo. **(f)** The interactions between the G$_{+2}$(nt) and the 'G' pocket are the same in *Mtb* σ^{H}-RPo (pink) and *Mtb* σ^{A}-RPo (gray; PDB: 5UHA). **(g)** The interaction of the RNA/DNA hybrid with σ$^{H}_{3.2}$ and RNAP core enzyme in σ^{H}-RPo. **(h)** The interaction of RNA/DNA hybrid with σ$^{A}_{3.2}$ and RNAP core enzyme in *Mtb* σ^{A}-RPo (PDB: 5UHA). **(i)** The σ$^{A}_{3.2}$ in *Mtb* σ^{A}-RPo (gray; PDB: 5UHA) reaches deeper into the active center compared with σ$^{H}_{3.2}$ in σ^{H}-RPo (pink). Green mesh, simulated-annealing Fo-Fc difference map with nucleic acids omitted contoured at 2.5 σ.
