# Supplementary material for: Structural basis for transcription initiation by bacterial ECF σ factors
Source: Nat Commun. 2019 Mar 11;10:1153. doi: 10.1038/s41467-019-09096-y (PMC6411747; doi:10.1038/s41467-019-09096-y)
Supplement: Supplementary file 3 — Description of Additional Supplementary Files [file 41467_2019_9096_MOESM3_ESM.pdf]

**Description of Additional Supplementary Files**

File Name: Supplementary Data 1
Description: The sequences of primers used in the study.
